# Extra Fingers and Toes

**Published:** 1858

**Authors:** Wm. Gutch

**Affiliations:** Blakesburg, Iowa


### Case of Extra Fingers and Toes.

BY WM. GUTCH, M. D., OF BLAKESBURG, IOWA.

Mrs. W——, aged 38 years, the mother of several children was delivered on the 14th January, 1853, of a female child having six fingers on each hand, and the same number of toes on each foot. The extra fingers were pendulous, being attached on the ulnar side of the hand, opposite the metacarpo-phalangeal articulation of the little finger, by a sort of pedicle composed of integument, blood vessels, &c. The extra toes have the same direction as the others; but as there is no metatarsal bone, they are articulated with the little toes. As the supernumerary fingers would have been both inconvenient and unsightly, I passed a ligature round the pedicle of each and removed them with the scissors.

In February, 1855, Mrs. W.——— was delivered of a male child, about which there was nothing remarkable. On the 23d July, 1857, Mrs. W. was delivered of another female child, having the extra fingers, as in the case first mentioned, but having the proper number of toes. I amputated these fingers also. Both children are now living. The fingers I still have in my possession. WM. GUTCH, M. D.

